# Teriflunomide Does Not Change Dynamics of Nadph Oxidase Activation and Neuronal Dysfunction During Neuroinflammation

**DOI:** 10.3389/fmolb.2020.00062

**Published:** 2020-04-30

**Authors:** Ronja Mothes, Carolin Ulbricht, Ruth Leben, Robert Günther, Anja E. Hauser, Helena Radbruch, Raluca Niesner

**Affiliations:** ^1^Institute for Neuropathology, Charité Universitätsmedizin Berlin, Berlin, Germany; ^2^Deutsches Rheumaforschungszentrum – Leibniz Institute, Berlin, Germany; ^3^Immunodyanmics and Intravital Microscopy, Charité Universitätsmedizin Berlin, Berlin, Germany; ^4^Veterinary Medicine, Freie Universität Berlin, Berlin, Germany

**Keywords:** teriflunomide, EAE, oxidative stress, neuronal dysfunction, calcium imaging

## Abstract

The multiple sclerosis therapeutic teriflunomide is known to block the *de novo* synthesis of pyrimidine in mitochondria by inhibiting the enzyme dihydroorotate-dehydrogenase (DHODH). The metabolic processes of oxidative phosphorylation and glycolysis are further possible downstream targets. In healthy adult mice, high levels of dihydroorotate-dehydrogenase (DHODH) activity are measured in the central nervous system (CNS), and DHODH inhibition may cause indirect effects on reactive oxygen species production and NADPH oxidase (NOX) mediated oxidative stress, known to be key aspects of the inflammatory response of the CNS. However, little is known about the effect of teriflunomide on the dynamics of NOX activation in CNS cells and subsequent alterations of neuronal function *in vivo*. In this study, we employed fluorescence lifetime imaging (FLIM) and phasor analysis of the endogeneous fluorescence of NAD(P)H (nicotinamide adenine dinucleotide phosphate) in the brain stem of mice to visualize the effect of teriflunomide on cellular metabolism. Furthermore, we simultaneously studied neuronal Ca^2+^ signals in transgenic mice with a FRET-based Troponin C Ca^2+^ sensor based (CerTN L15) quantified using FRET-FLIM. Hence, we directly correlated neuronal (dys-)function indicated by steadily elevated calcium levels with metabolic activity in neurons and surrounding CNS tissue. Employing our intravital co-registered imaging approach, we could not detect any significant alteration of NOX activation after incubation of the tissue with teriflunomide. Furthermore, we could not detect any changes of the inflammatory induced neuronal dysfunction due to local treatment with teriflunomide. Concerning drug safety, we can confirm that teriflunomide has no metabolic effects on neuronal function in the CNS tissue during neuroinflammation at concentrations expected in orally treated patients. The combined endogenous FLIM and calcium imaging approach developed by us and employed here uniquely meets the need to monitor cellular metabolism as a basic mechanism of tissue functions *in vivo*.

## Introduction

Current treatment modalities of neuroinflammation are targeting the activation of immune cells, or their migration into inflamed tissue and do not prevent neurodegeneration when patients once entered the chronic progressive phase. On the other hand, it is clear that the immune system drives chronic neuroinflammation, since immunoablation in combination with autologous hematopoietic stem cell transplantation can induce remission in refractory relapsing and remitting multiple sclerosis patients (Burt et al., 2015). However, in contrast to patients with relapses, patients in the chronic phase of secondary or primary progressive multiple sclerosis lack overt inflammation, despite ongoing neuronal damage. Then a modulation of central nervous system (CNS) resident cells is needed to enhance repair and minimize progression.

After first being used in rheumatic diseases against transplant reactions, teriflunomide, the active metabolite of leflunomide was approved for relapsing multiple sclerosis as an immune modulatory treatment by the US Food and Drug Administration and European Medicine Agency. The primary mode of action of teriflunomide is contributed to the inhibition of the proliferation of lymphocytes. During the (pathological) activation, lymphocytes enter the cell cycle and need for the DNA synthesis pyrimidine and purine bases. It has been shown that Teriflunomide blocks *de novo* pyrimidine synthesis by specific non-competitive, reversible inhibition of the mitochondrial enzyme dihydro-orotate dehydrogenase (DHODH) (Cherwinski et al., 1995; Williamson et al., 1995). But in addition, DHODH inhibition corrects the metabolic disturbances in T cells, which primarily affects metabolically active high-affinity T cell clones, shaping the affinity spectrum (Klotz et al., 2019).

DHODH is not only expressed at high levels in proliferating lymphocytes, but also in cells of the CNS (Schaefer et al., 2010; Wostradowski et al., 2016). Furthermore, teriflunomide can cross the blood-brain barrier with 1–2% of serum concentrations (in the range of 2.5–4.1 μM) (Wiese et al., 2013). Moreover, metabolic changes in the CNS associated with the presence of teriflunomide’s presence were detectable (Rzagalinski et al., 2019). And besides the described hypothesized main modes of action by inhibition of DHODH in activated lymphocytes, *in vitro* data suggests additional effects of teriflunomide as a modulator of Ca^2+^ signaling (Rahman and Rahman, 2017), NF-kB and related signaling pathways (Manna and Aggarwal, 1999; Göttle et al., 2018) and also cytokine production in monocytes (Li et al., 2013), which represent the main infiltrating celltype during neuroinflammation.

As cytosolic reactive oxygen species are formed most notably through NADPH oxidases (NOX) activity and influence metabolic processes including glycolysis and downstream oxidative phosphorylation (Forrester et al., 2018), inhibition of DHODH is also discussed to have an influence on NOX activity mainly in the context of cancer e.g., in transformed skin cells, ROS production was reduced after incubation with teriflunomide (Hail et al., 2010). These processes of NOX alteration via DHODH inhibition and the other way round could be important not only in cancer but also during autoimmunity. In addition, mitochondria are a possible source of ROS via their DHODH activity, which then can stimulate NOX. Targeting this crosstalk between metabolism and NOX is expected to be pharmaceutical relevant especially under oxidative stress conditions as present in diseases like multiple sclerosis in the CNS. Under this aspect the mechanism of modulated cellular energetics by teriflunomide (Klotz et al., 2019) could lead to an antioxidant effect of teriflunomide directly in the CNS. This could contribute to neuroprotection and would be relevant especially in the progressive phase, when the presence of lymphocytes in the CNS is minimal. On the other hand, pro-oxidative effects and impairing the metabolic capacity of CNS cells would be detrimental to neuronal function. To tackle this question, new experimental approaches are needed as conventional *in vivo* imaging setups cannot report the metabolic state and interactions of cells in neuroinflammatory lesions.

We employed here an intravital imaging approach which combines NAD(P)H-FLIM – as a measure of metabolic activity – with FRET-FLIM, to measure absolute neuronal calcium concentrations, as first signs of neuronal dysfunction (explained in more detail in the methods section). By employing our combined NAD(P)H-FLIM and Calcium FRET-FLIM approach in the brain stem of CerTN L15 mice and CX_3_CR1:GFP mice, respectively, we were able to determine drug-induced changes in the metabolic state in health and during the inflammatory process at the lesion site and access their impact on the neuronal (dys)function *in vivo*. Thus, we could not detect any significant metabolic alteration in the tissue after local short-term incubation with teriflunomide – neither in health nor in neuroinflammation.

## Materials and Methods

### Two-Photon Microscopy Setup for Fluorescence Lifetime Imaging

**FIGURE 1:**
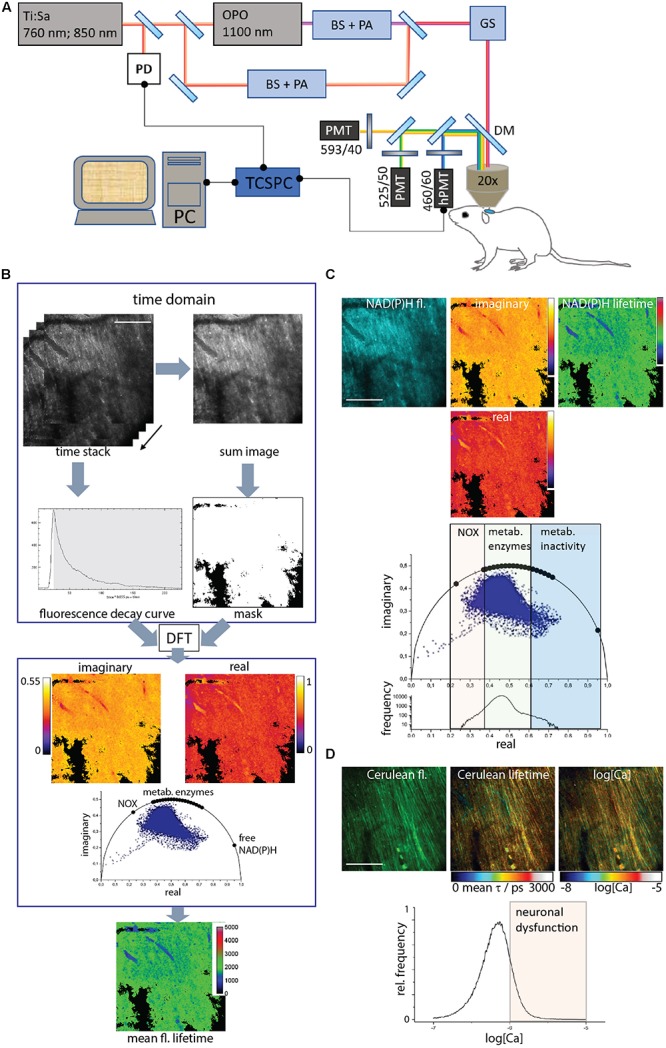
Acquisition and evaluation of Cerulean and NAD(P)H fluorescence lifetime imaging data in brain stem tissue. **(A)** Experimental setup. A part of the beam of a 80 MHz pulsed titanium sapphire laser (Ti:Sa) at 760 or 850 nm passes a beam shaper, including a telescope and pulse compressor, and a λ/2 plate-based power attenuator (PA). The other part of the beam (850 nm) pumps the optical parametric oscillator (OPO). The OPO beam also passes a beam shaper and a power attenuator (PA), similar to the Ti:Sa beam. The Ti:Sa and OPO beams are combined through a dichroic mirror. A galvoscanner (GS) scans the combined beam over the sample: the brain stem of an anesthetized mouse. The laser beams are focused by a 20 × water immersion NA 1.05 objective. Excitation and emission light are separated by a dichroic mirror (DC, 695 nm), the emission light passes an interference filter (IF 466/40) and is time-resolved detected by a time-correlated single photon counter (TCSPC) equipped with a Hybrid-PMT (h-PMT). A beam splitter reflects 5–10% of the laser light onto a photodiode (PD) to synchronize the TCSPC in time. The additional emitted fluorescence is analyzed by a series of dichroic mirrors and interference filters and detected by PMTs. (B) Data acquisition and evaluation. The TCSPC raw data contains 227 images. In each pixel, a typical time-domain decay curve is measured. A binary mask is created by thresholding the sum image of the single images to exclude background pixels. Each pixel in the raw data set is converted by discrete Fourier transform to the phase domain at 80 MHz modulation frequency. Each pixel contains a complex number with a real and an imaginary part, which give the coordinates of the phase vector (= phasor). Their 2D-distribution results into the phasor plot. The phasors of free NAD(P)H, NAD(P)H bound to metabolic enzymes and NADPH bound to NADPH oxidases are labeled on the half circle. By back-transforming the real and imaginary images, a mean fluorescence lifetime image is calculated. **(C)** The time-resolved NAD(P)H fluorescence image is transformed to the phase domain into a real, an imaginary image and the phasor plot. By back-transforming the phase-domain data, the mean NAD(P)H fluorescence lifetime image is generated. Both in the phasor plot and in the histogram of the real part of the phasor vector, the NOX enzyme activation, metabolic activity and low enzymatic activity regions can be resolved. **(D)** The time-resolved Cerulean fluorescence data lead, as described in **(B)**, to the mean unquenched and FRET-quenched Cerulean fluorescence lifetime image. Relying on the *K*_*d*_ and *Hill slope* of the Calcium-sensitive FRET-sensor TN L15, a calcium concentration image is generated from the mean Cerulean fluorescence lifetime image. In the histogram of neuronal Calcium concentration (log[Ca]), the neuronal dysfunction regime is indicated by concentrations higher than 1 μM or a log[Ca] = –6. Scale bars represent 100 μm.

Experiments were carried out using a specialized two-photon laser-scanning microscope for fluorescence lifetime imaging (FLIM) as displayed in Figure 1A. In brief, the beam of a tuneable fs-pulsed Ti:Sa laser (wavelength range 700–1,080 nm, 140 fs, 80 MHz, Cameleon Ultra II, Coherent, Dieburg, Germany) is combined with the tuneable fs-pulsed optical parametric oscillator (Compact OPO pumped by Ti:Sa, 1,050–1,600 nm, 200 fs, 80 MHz, APE, Berlin, Germany). Both beams are scanned by two galvanometric mirrors and focused into the sample by an objective lens for deep-tissue imaging (20 × dipping lens, NA 1.05, WD 1 mm-Zeiss, Jena, Germany). The resulting fluorescence signal is detected and analyzed either by a TCSPC point detector (FLIM, LaVision Biotech GmbH, Bielefeld, Germany) and photomultiplier tubes (H7422-40, Hamamatsu, Japan). Spectral discrimination of the fluorescence signal was achieved using appropriate dichroic mirrors and interference filters. NADH and NADPH were excited at 760 nm and Cerulean – donor in the TN L15 FRET-construct – at 850 nm. Both molecules were detected through an interference filter at 466/40 nm. The time step (bin) was 55 ps and the time window for measuring the fluorescence decay was 12.4 ns. tdRFP in LysM^+^ cells was excited at 1,100 nm of the OPO and detected at 593/40 nm. eGFP in CX_3_CR1^+^ cells was excited at 920 nm and detected at 525/50 nm.

### Mouse Strains

All mice used were on a C57/Bl6 background. The *CerTN L15 x LysM tdRFP* mouse expresses a FRET-based calcium biosensor consisting of Cerulean (donor) and Citrine (acceptor) bound to troponin C, a calcium-sensitive protein present in certain subsets of neurons. Mice carrying the CerTN L15 reporter were crossed to the LysM tdRFP mice, in which tdRFP is expressed in LysM + cells, yielding progeny with both reporters (Mossakowski et al., 2015). The CX3CR1 ± :eGFP mouse was used to detect microglia.

### Experimental Autoimmune Encephalomyelitis

Experimental autoimmune encephalomyelitis (EAE) was induced as described previously (Radbruch et al., 2016). Briefly, mice were immunized subcutaneously with 250 μg of MOG35–55 (Pepceuticals, United Kingdom) emulsified in CFA (BD Difco, Germany) and received 400 ng pertussis toxin (PTx, List Biological Laboratories, Inc.) intraperitoneally at the time of immunization and 48 h later. Intravital multi-photon microscopy was carried out at the peak of disease.

### Brain Stem Preparation for Imaging

The brain stem was exposed by carefully removing the musculature above the dorsal neck area and removing the dura mater between the first cervical vertebra and the occipital skull bone. The head was inclined for access to deeper brainstem regions and the brain stem superfused with isotonic Ringer solution. Anesthesia depth was controlled by continuous CO_2_ measurements of exhaled gas and recorded with a CI-240 Microcapnograph (Columbus Instruments, United States) and by an Einthoven three-lead electrocardiogram (ECG). In order to avoid strong breathing artifacts in the brainstem of anesthetized mice, the ECG signal was correlated to the respiration rate and used as an external trigger for the image acquisition software, which controls the hardware of the microscope setup. Thus, each fluorescence stack was recorded in the same tissue region at the same point in the respiratory cycle of the mouse. Respiratory function, heart frequency and body temperature were checked continuously during operation and imaging. In each imaged animal, the whole area that was accessible by intravital imaging was scanned and all visible lesions of each animal were analyzed as well as at least one “normal appearing” imaging field. For the teriflunomide treatment, 500 μL of 5 μM teriflunomide solution was locally applied to the brain stem of healthy or diseased mice similar to previous experiments with glutamate (Mossakowski et al., 2015). Teriflunomide was provided in powder form by the manufacturer (Sanofi), which was dissolved in DMSO and stored at –20°C. Animal experiments were approved by the appropriate state committees for animal welfare (LAGeSo—Landesamt für Gesundheit und Soziales Berlin) and were done in accordance with current guidelines and regulations.

### The Phasor Approach to FLIM

The FLIM data are measured in time domain and transferred to a virtual phase domain by numerically calculating the discrete Fourier transformation. The sum of all fluorescence lifetimes contained in a pixel is calculated from the normalized real and imaginary part. In case of a mono-exponential decay, plotting those results will give a position on a half-circle [*r* = 0.5, center at (0.5/0)], which give the coordinates of the phase vector (= phasor). If the excitation volume contains two fluorescent species, this position in the plot will lie along the straight line connecting the phasors of the pure components. If there are three species, this position would lie within the triangle formed by the line connecting the phasors of the pure components. The phasor analysis was performed using our own routines written in Python.

### Validation of NAD(P)H-FLIM in CerTN L15 Mice

We demonstrated that by applying the phasor approach to NAD(P)H-FLIM data we can determine metabolic activity levels as well as preferential enzymatic activity in cells and tissues. Thereby, we are able to resolve between diverse abundant NAD(P)H-dependent enzymes such as lactate dehydrogenase, malate dehydrogenase, GAPDH, pyruvate dehydrogenase or NOX (Leben et al., 2019). Using our approach, we were able to show that the preferential activation of NOX accompanies phagocytosis and precedes NETosis in myeloid cells (Leben et al., 2018). In diverse tissue of fluorescence reporter mice we could previously confirm that under excitation at 760 nm (pulse width ≈140 fs, repetition rate 80 MHz) we mainly detect the fluorescence of the coenzymes NADH and NADPH and not the genetically encoded fluorescent proteins. Hence, we validated the purity of our NAD(P)H signal by performing experiments at 4°C as compared to 37°C in brain slices of CerTN L15 mice which express the FRET-based Ca^2+^ biosensor TN L15 in certain neuronal subsets (Heim et al., 2007). The TN L15 sensor is based on Cerulean (cyan fluorescent) and Citrine (yellow fluorescent) as a FRET pair bound to Troponin C – a calcium sensitive protein. Additionally, it is generally known that the co-enzymes NAD(P)H can bind to enzymes at 37°C, but the binding affinity decreases with decreasing temperature. On ice (at 4°C), NAD(P)H do not bind to enzymes. Referring to the fluorescence lifetime of NAD(P)H, it is known that free NAD(P)H has a short fluorescence lifetime (in average 450 ps) and, consequently shows a low signal, whereas enzyme-bound NAD(P)H has much longer fluorescence lifetimes (in the range of 1,000 ps to as high as 5,000 ps) and consequently displays a much higher fluorescence signal. At 4°C in brain slices of CerTN L15 mice we could not detect any cell-specific signal, whereas at 37°C there is clear evidence of fluorescence originating from cells which does not coincide with the fluorescence signal of Cerulean (from the TN L15 sensor expressed in neurons) detected in the same samples under excitation at 850 nm (Heim et al., 2007; Radbruch et al., 2015). Furthermore, the fluorescence signal under excitation at 760 nm dramatically increased in the samples after adding NaCN to then decrease after cell death (Niesner et al., 2008; Mossakowski et al., 2015). We have done similar observations in GFP reporter mice such as the CX3CR1:GFP mice (Mossakowski et al., 2015). Thus, we could conclude from our previous work that under excitation at 760 nm, we will detect only endogenous NAD(P)H fluorescence in the 460/60 nm detection channel, even in cells and tissues expressing fluorescent proteins (such as Cerulean or GFP). We assume that in cells, the low expression of fluorescent proteins as compared to the coenzymes NADH and NADPH – needed for cell survival – counteracts the much lower two-photon absorption cross-sections of the endogenous NAD(P)H as compared to the fluorescent proteins (typically several orders of magnitude; Zipfel et al., 2003; Rinnenthal et al., 2013).

Moreover, the Cerulean fluorescence signal detected in the 460/60 nm channel from brain slices of CerTN L15 mice, under excitation at 850 nm, did not change when we shifted the temperature from 37 to 4°C, thus, indicating no measurable contribution of NAD(P)H to the fluorescence signal. Under these conditions, we conclude that we will detect only Cerulean fluorescence, the decay of which we measured by means of FLIM to determine the sub-cellular FRET efficiency of TN L15 in the cytosol of neurons (Heim et al., 2007). We could previously demonstrate that measuring FRET efficiency by FLIM deep within tissues is reliable in contrast to ratiometric FRET-measurements which are prone to artifacts due to different emission scattering and photobleaching properties of the donor as compared to those of the acceptor (Radbruch et al., 2015). By measuring the FRET efficiency by FRET-FLIM of TN L15 in solutions of different free calcium concentrations, we provided a titration curve of the biosensor which allows us to directly translate FRET efficiencies into absolute calcium values (Rinnenthal et al., 2013).

In addition we could previously show that elevated neuronal calcium levels in the brain stem of mice (both healthy mice locally treated with KCl or ionomycine and mice affected by EAE) lead after 2 h to axonal disruption and finally to neuronal death (Herz et al., 2010; Siffrin et al., 2010; Rinnenthal et al., 2013; Mossakowski et al., 2015; Radbruch et al., 2016, 2017). Thus, elevated neuronal calcium beyond 1 μmol/L directly indicates neuronal dysfunction also in CNS tissue, in line with reports which demonstrated death of neurons at sustained 1 μmol/L Ca^2+^ concentration in primary cell cultures (Schlaepfer and Zimmerman, 1985).

## Results

### Correlating Neuronal Dysfunction and NAD(P)H-Dependent Enzymatic Activity Responsible for Oxidative Stress Generation by the Phasor Approach to FLIM

We carried out intravital FLIM in the brain stem of *CerTN L15xLysM:tdRFP* and *CX3CR1:eGFP* mice, in health and during experimental autoimmune encephalomyelitis (EAE). In this way, we assessed the NAD(P)H-dependent enzymatic activity leading to oxidative stress generation, i.e., over-activation of NOX, as well as neuronal dysfunction indicated by neuronal calcium over 1 μM.

We employed time-domain FLIM by time-correlated single-photon counting (TCSPC) in a specialized two-photon microscope (Figure 1A) to acquire the fluorescence decay curve of either the coenzymes NADH or NADPH, i.e., NAD(P)H, excited at 760 nm and detected at 466/40 nm or of Cerulean – the donor in the Calcium-sensitive FRET-construct TN L15 – excited at 850 nm and detected also at 466/40 nm.

We evaluated all data using the phasor approach to FLIM (Figure 1B). The time-resolved data were discretely Fourier-transformed into the frequency (phase) domain. Thereby, in each pixel of the image a phasor vector was generated, i.e., a complex number with a real and an imaginary part. The histogram of all pixel phasor vectors in an image are displayed in the phasor plot. By Fourier back-transforming the real and the imaginary part images, a mean fluorescence lifetime image was generated (Figure 1B).

By employing NAD(P)H-FLIM, we monitored the NAD(P)H-dependent enzymatic activity of all cells in the brain stem of living mice. Free NAD(P)H is characterized by a short fluorescence lifetime (450 ps), whereas enzyme-bound NAD(P)H is characterized by a much longer fluorescence lifetime – depending on the NAD(P)H binding site on the enzyme, between 1,200 and 3,650 ps (Figure 1C). Both the phasor plot and the histogram of the real part of the phasor vector are able to distinguish between NOX enzyme activation, normal metabolic activity and low metabolic activity as shown in Leben.

The TN L15 FRET-construct is based on the calcium sensitive muscle protein Troponin C coupled to Cerulean as a FRET-donor and Citrine as a FRET-acceptor. The sensor has two high-affinity and two low affinity Ca-binding sites, with a *K*_*d*_ of 1.2 μM and a *Hill slope* of 1.46. In the *CerTN L15* mouse strain, the construct is expressed in certain neuronal subsets. By applying the phasor approach to FRET-FLIM in the brain stem of *CerTN L15* mice we determined the mean fluorescence lifetime of Cerulean, combining both FRET-quenched and unquenched states. Using the previously measured calibration curve (Rinnenthal et al., 2013) we determined the absolute Calcium concentration in neurons (Figure 1D). As shown in Figures 1C,D, correlative measurements of NAD(P)H enzymatic activity and of neuronal Calcium are feasible in the brain stem of *CerTN L15* mice.

### Correlative NAD(P)H-FLIM and FRET-FLIM Distinguishes Between Non-lesion and Lesion Sites in EAE

**FIGURE 2:**
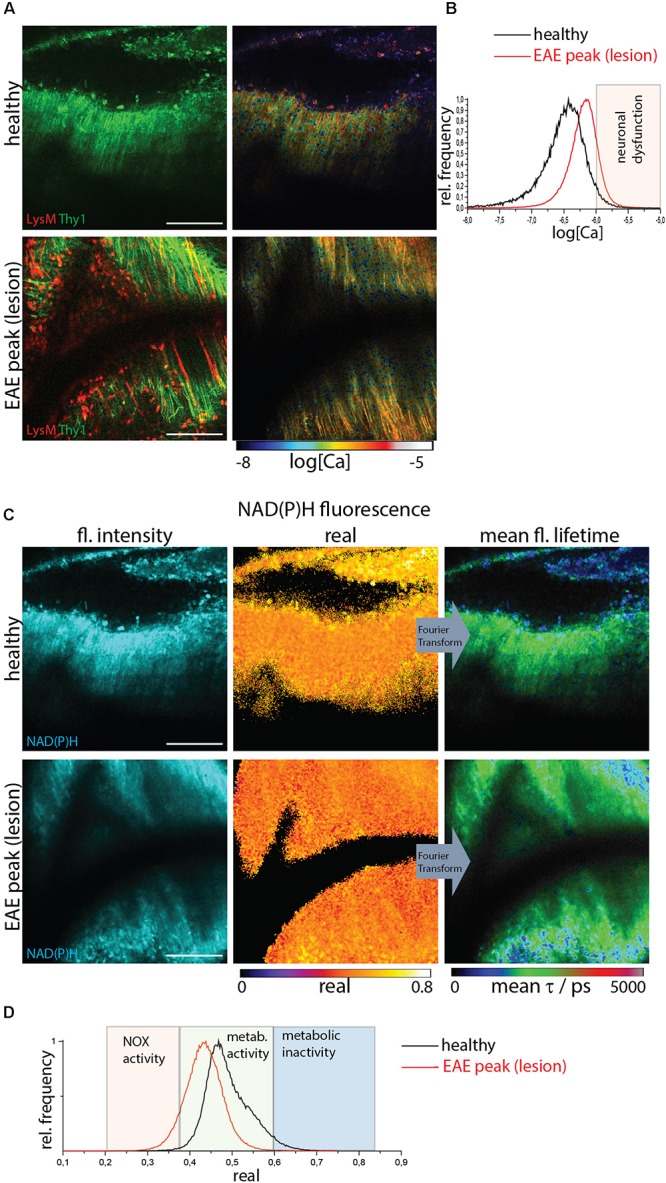
NOX enzymes activation by NAD(P)H-FLIM and neuronal dysfunction by FRET-FLIM of TN L15 in the brain stem of healthy vs. EAE mice. **(A)** FRET-FLIM as a measure for neuronal dysfunction. Fluorescence intensity images of a healthy (upper image) and diseased (lower) brain stem of *CerTN L15 x LysM tdRFP* mice: tdRFP fluorescence in LysM^+^ cells (red) and Cerulean and Citrine fluorescence in Thy 1 cells (green). Corresponding log[Ca] images of the regions shown by the fluorescence intensity images. **(B)** Histograms of neuronal Calcium in log[Ca] depicting the regime of neuronal dysfunction in the images in **(A)**. **(C)** NAD(P)H fluorescence intensity images, real part images and mean NAD(P)H fluorescence lifetime images of the same a healthy (upper image) and diseased (lower) brain stem of *CerTN L15 x LysM tdRFP* mouse as in **(A)**. **(D)** Histograms of the real part of the NAD(P)H phasor vector depicting the NOX enzymes activation regime in the images in **(C)**. **(A–D)** Are representative data for *n* = 2 healthy mice and *n* = 3 mice affected by EAE in peak of the disease. Scale bars represent 100 μm.

We identified lesion sites using two-photon microscopy in the brain stem of *CerTN L15xLysM:tdRFP* mice affected by EAE based on the presence of LysM^+^ cell infiltration. Infiltration-free regions are typical for healthy mice but can also be found in diseased mice (Figure 2A).

FRET-FLIM of the TN L15 construct (Figure 2A) allowed us to show that in healthy mice or lesion-free regions of diseased mice, the mean neuronal Calcium concentration does not exceed 1 μM, whereas in regions with immune infiltration ∼10% of the neurons have a calcium concentration over 1 μM and, thus, show neuronal dysfunction (Figure 2B). NAD(P)H-FLIM of the same regions revealed that NOX enzymes activation correlates with immune infiltration and high neuronal calcium (Figure 2C), as shown by the histograms of the real part of the phasor vector (Figure 2D).

### Teriflunomide Does Not Lead to Increased NOX Activity or Neuronal Calcium in Healthy Mice

**FIGURE 3:**
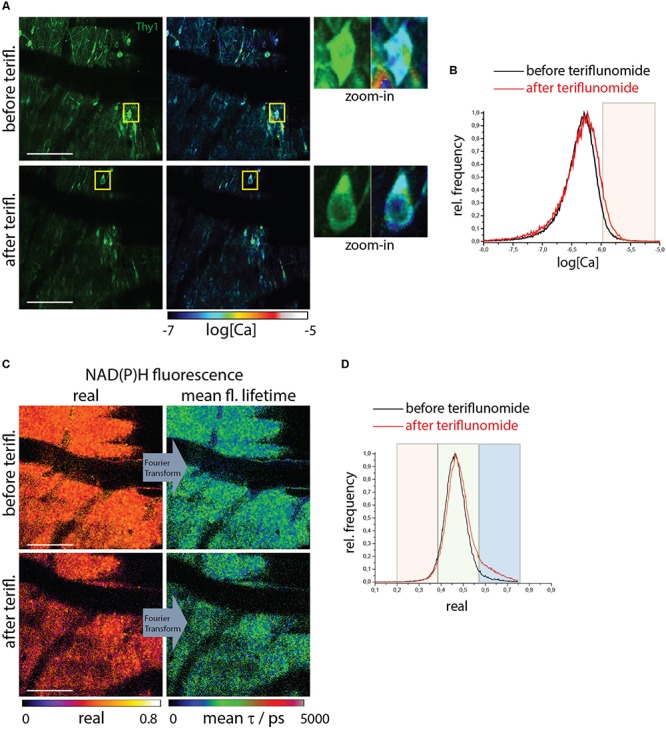
NOX enzymes activation by NAD(P)H-FLIM and neuronal dysfunction by FRET-FLIM of TN L15 in the brain stem of healthy mice under treatment with teriflunomide. **(A)** Fluorescence intensity images of an untreated (upper image) and treated (lower) brain stem of healthy *CerTN L15 x LysM tdRFP* mice: Cerulean and Citrine fluorescence in Thy 1 cells (green). Corresponding log[Ca] images of the regions shown by the fluorescence intensity images. Yellow frames depict the regions of untreated and treated neurons, respectively, displayed enlarged. **(B)** Histograms of neuronal Calcium in log[Ca] with and without teriflunomide treatment. **(C)** NAD(P)H fluorescence real part images and mean NAD(P)H fluorescence lifetime images of the same untreated (upper image) and treated (lower) brain stem region of the *CerTN L15 x LysM tdRFP* mouse as in **(A)**. **(D)** Histograms of the real part of the NAD(P)H phasor vector depicting the NAD(P)H-dependent enzymes activation in the images in **(C)**. **(A–D)** Are representative data for *n* = 2 healthy mice (Supplementary Table S1). Scale bars represent 100 μm.

We used healthy mice as a negative control without any pre-existing NOX overactivation and pathological calcium as showed above. We carried out correlative FRET-FLIM and NAD(P)H-FLIM in the brain stem of healthy *CerTN L15xLysM:tdRFP* mice before and 5 min after local application of teriflunomide. Both the neuronal calcium (Figures 3A,B) and the NAD(P)H-dependent enzymatic activity (Figures 3C,D) were not changed by teriflunomide and stayed at low levels (Supplementary Table S1) in contrast to local short-term application of glutamate (Mossakowski et al., 2015).

### Teriflunomide Do Not Counteract Increased NOX Activity and Neuronal Dysfunction in the Lesions of EAE Mice

Lesions of mice affected with EAE served as positive controls with already preexisting NOX overactivation and increase in neuronal calcium levels. We performed correlative FRET-FLIM and NAD(P)H-FLIM in the brain stem of *CerTN L15xLysM:tdRFP* and *CX_3_CR1:eGFP* mice affected by EAE, in the peak of the disease, before and 5 min after local application of teriflunomide.

**FIGURE 4:**
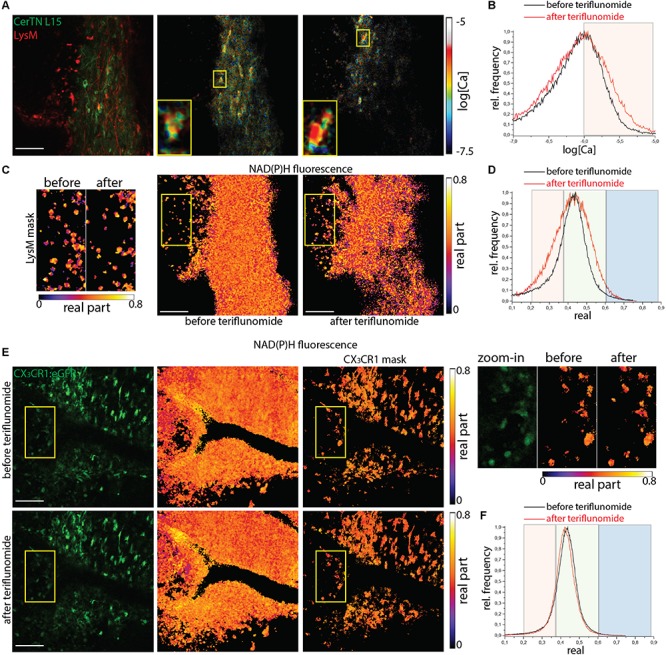
NOX enzymes activation by NAD(P)H-FLIM and neuronal dysfunction by FRET-FLIM of TN L15 in the brain stem of mice affected by EAE under treatment with teriflunomide. **(A)** Fluorescence intensity image and corresponding log[Ca] images of the same region in the brain stem of a *CerTN L15 x LysM tdRFP* mouse affected by EAE (score 2.5), before and after teriflunomide treatment: tdRFP fluorescence in LysM^+^ cells (red) and Cerulean and Citrine fluorescence in Thy 1 cells (green). Insets depict single untreated and treated neurons, respectively, displayed enlarged. **(B)** Histograms of neuronal Calcium in log[Ca] with and without teriflunomide treatment. **(C)** NAD(P)H fluorescence real part images of the same untreated and treated region in the brain stem of the *CerTN L15 x LysM tdRFP* mouse as in **(A)**. Enlarged images of the real part of the NAD(P)H fluorescence phasor vector in LysM^+^ cells, before and after treatment. **(D)** Histograms of the real part of the NAD(P)H phasor vector depicting the NOX enzymes activation regimes in the overview images in **(C)**. **(E)** eGFP fluorescence intensity images and NAD(P)H fluorescence real part images of the same untreated and treated region in the brain stem of a *CX_3_CR1:eGFP* mouse affected by EAE (score 3.0), at a lesion site. Upper raw display the NAD(P)H in all cells, the lower raw the NAD(P)H in CX_3_CR1^+^ cells. Yellow frames depict the regions of phagocyte-shaped CX_3_CR1^+^ cells displayed enlarged (right side). **(F)** Histograms of the real part of the NAD(P)H phasor vector depicting the NOX enzymes activation regimes in the overview images in **(E)**. Scale bars represent 100 μm.

In *CerTN L15xLysM:tdRFP* mice, both the neuronal calcium (Figures 4A,B) and the NAD(P)H-dependent enzymatic activity (Figures 4C,D) stayed at high levels despite teriflunomide application (Supplementary Table S1). As shown in the zoom-in images, calcium concentration remains high before and after treatment in single neurons. The NOX enzymes activation in LysM^+^ cells (monocytes and macrophages) responsible for oxidative stress generation in the CNS during EAE is also not reduced by the local treatment with teriflunomide and the cells show unaltered morphology. In *CX_3_CR1:eGFP* mice at peak of EAE, the NOX activation in gliotic areas characterized by phagocyte-shaped CX_3_CR1^+^ cells stays at high levels before and after application of teriflunomide. This hold true both in all cells of the tissue and, specifically, in CX_3_CR1^+^ cells (Figures 4E,F).

## Discussion

Our results support the understanding of the direct effect of teriflunomide on CNS cells, especially regarding oxidative stress and neuronal dysfunction. Teriflunomide is used as an immunomodulatory treatment in patients with relapse and remitting multiple sclerosis since several years. It has a well characterized safety and tolerability profile (Bar-Or et al., 2014). Phase III studies showed significant reductions in disability progression, relapse rates, and magnetic resonance imaging measures of disease activity (O’Connor et al., 2011). Teriflunomide’s main mode of action is selective inhibition of activated T and B lymphocyte proliferation. For this effect only very low concentrations in a range of 600 nM are needed (Fox et al., 1999). Interestingly, additional effects at very high concentrations (>50 μM) (Williamson et al., 1995; Xu et al., 1995) and with special regimens as short-term pulses have been described (Göttle et al., 2018).

Despite these promising results at high dosages or with special regimens we preferred to use dosages adapted to the expected low concentrations in the CNS in patients under oral treatment to analyze the effect of teriflunomide in the CNS. In order to evaluate further mechanisms of action or side effects of teriflunomide as modulation of the Ca^2+^ signaling (Rahman and Rahman, 2017), NF-kB and related signaling pathways (Manna and Aggarwal, 1999; Göttle et al., 2018), oxidative stress and cellular metabolism and its interaction (Hail et al., 2010; Klotz et al., 2019) in the CNS, we investigated its effects on oxidative stress and neuronal calcium level in MOG_35__–__55_ induced EAE affected mice after local application on the brainstem *in vivo*. Using the approach, we could circumvent the indirect effect on CNS cells by the action of teriflunomide on lymphocytes as we applied the substance directly on the lesioned CNS.

Oxidative stress by overproduction of ROS by CNS resident cells is one major component of neuronal damage especially in the progressive phase of EAE (Radbruch et al., 2016), when the peripheral immune cell infiltration disappeared. But ROS *per se* are highly reactive and it is difficult to quantify them *in vivo* in the tissue. The endogenous FLIM approach presented here uniquely meets the increasing need in the field of neuroimmunology to monitor cellular metabolism as a basic mechanism of tissue functions *in vivo* (Bayerl et al., 2016). The ubiquitous coenzymes NADH and NADPH in cells show a short fluorescence lifetime (~400 ps) in the free−state and a longer fluorescence lifetime when bound to enzymes. In the case of NADPH bound to members of the NOX family 3,650 ps are measured which can safely be distinguish from enzymes typically active in the cytoplasm with fluorescence lifetimes of ∼2,000 ps. Using NAD(P)H FLIM enables to monitor the catalytic function of ROS production i.e., the activity of NOX family members in different pathologies as tumor, degeneration and inflammation (Bremer et al., 2016; Radbruch et al., 2016, 2017).

Concerning drug safety, there was no reduced viability of the mice during the anesthesia. We monitored heart frequency, body temperature and respiratory function. These parameters were all unchanged after short term local application of teriflunomide. Additionally, we could not detect any altered tissue integrity, morphology and function, nor was there altered vessel permeability and/or edema. Our experiments confirm that teriflunomide is not additionally inducing NOX activation, i.e., oxidative stress in the CNS tissue *in vivo* during EAE nor under healthy uninflamed conditions. The present findings suggest no potential neurotoxic side effects but also no additional short-term neuroprotective effect measurable with our tools, knowing that we can only detect high and sustained levels of calcium and NOX activity that are present during inflammation and not subtle changes of NOX enzyme activity nor fast calcium changes that can be observed in cell signaling.

We could not detect any changes on tissue activation state by this approach after application of teriflunomide during EAE and in healthy mice. We wanted to circumvent the effect of teriflunomide on lymphocytes and focus on the CNS compartment during neuroinflammation to observe the very same area with and without teriflunomide in the same animal. Therefore, a longer systemic (pre)treatment of the animals would make an isolated view on the CNS compartment impossible.

As quantification of NOX activity can be used as a surrogate marker for phagocytic activity (Mossakowski et al., 2015) our results are in line with previous reports on microglia cultures which showed no enhancing or suppressive effects of teriflunomide on phagocytosis (Wostradowski et al., 2016) with similar dosages *in vitro*.

Our results underline the need of intravital analysis for drugs with proposed mode of action on CNS cells as only in the tissue context CNS cell function and metabolic state due to interactions of cells in neuroinflammatory lesions can be analyzed.

## Data Availability Statement

The datasets generated for this study are available on request to the corresponding author.

## Ethics Statement

The animal study was reviewed and approved by Landesamt für Gesundheit und Soziales Berlin (LAGeSo) Turmstr. 21 10559 Berlin Germany.

## Author Contributions

RN, RM, AH, and HR planned the study and designed the experiments. RM, RG, RL, and CU performed the experiments. RL programmed phasor algorithm, RN performed the statistical analysis. RN, RM, and HR wrote the manuscript. RN and HR supervised the work. All authors provided input to the manuscript and approved the final draft.

## Conflict of Interest

The study was funded partly by financial support of Sanofi to HR and RN. The sponsor was not involved in the study design, data collection or analysis, drafting the manuscript, or the decision to publish. Furthermore, HR received speaker honoraria and research support from Novartis and Sanofi-Aventis/Genzyme unrelated to this project. The remaining authors declare that the research was conducted in the absence of any commercial or financial relationships that could be construed as a potential conflict of interest.
