# Supplementary material for: Teriflunomide Does Not Change Dynamics of Nadph Oxidase Activation and Neuronal Dysfunction During Neuroinflammation
Source: Front Mol Biosci. 2020 Apr 30;7:62. doi: 10.3389/fmolb.2020.00062 (PMC7203781; doi:10.3389/fmolb.2020.00062)
Supplement: Supplementary file 1 [file Table_1.docx]

Supplementary Material

## Supplementary Table 1

| mouse strain | disease condition | % area of neuronal calcium above 1 µM | | % area of NOX:  real in (0.2;0.38) | |
| --- | --- | --- | --- | --- | --- |
|  |  | before | after | before | after |
| *CerTN L15 x LysM:tdRFP* | healthy | 1.29% | 1.86% | 1.97% | 1.90% |
| *CerTN L15 x LysM:tdRFP* | healthy | 2.87% | 1.79% | 1.32% | 0.49% |
| *CerTN L15 x LysM:tdRFP* | EAE score 2.0 | 6.20% | 6.56% | 9.88% | 7.10% |
| *CerTN L15 x LysM:tdRFP* | EAE score 2.5 | 8.43% | 8.39% | 17.72% | 18.99% |
| *CerTN L15 x LysM:tdRFP* | EAE score 3.5 | 17.6% | 18.06% | 30.41% | 31.98% |
| *CX_3_CR1:eGFP* | EAE score 3.0 | -- | -- | 13.03% | 14.55% |

Mouse strains used in the experiments with standardized scores of the mice affected by experimental autoimmune encephalomyelitis and NADPH Oxidase activation before and after treatment with teriflunomide.
